# Lysine Methylation of the Valosin-Containing Protein (VCP) Is Dispensable for Development and Survival of Mice

**DOI:** 10.1371/journal.pone.0141472

**Published:** 2015-11-06

**Authors:** Markus Fusser, Stefan Kernstock, Vinay Kumar Aileni, Wolfgang Egge-Jacobsen, Pål Ø. Falnes, Arne Klungland

**Affiliations:** 1 Institute of Medical Microbiology, Oslo University Hospital, Rikshospitalet, Oslo, Norway; 2 Department of Biosciences, Faculty of Mathematics and Natural Sciences, University of Oslo, Oslo, Norway; 3 Glyconor Mass Spectrometry, Department of Biosciences, Faculty of Mathematics and Natural Sciences, University of Oslo, Oslo, Norway; 4 Institute of Basic Medical Sciences, University of Oslo, Oslo, Norway; Texas A&M University, UNITED STATES

## Abstract

Valosin-containing protein (VCP) is a homohexameric ATPase involved in a multitude cellular processes and it was recently shown that VCP is trimethylated at lysine 315 by the VCP lysine methyltransferase (VCPKMT). Here, we generated and validated a constitutive knockout mouse by targeting exon 1–4 of the *Vcpkmt* gene. We show that *Vcpkmt* is ubiquitously expressed in all tissues examined and confirm the sub-cellular localization to the cytoplasm. We show by (I) mass spectrometric analysis, (II) VCPKMT-mediated *in vitro* methylation of VCP in cell extracts and (III) immunostaining with a methylation specific antibody, that in *Vcpkmt* ^*-/-*^ mice the methylation of lysine 315 in VCP is completely abolished. In contrast, VCP is almost exclusively trimethylated in wild-type mice. Furthermore, we investigated the specificity of VCPKMT with *in vitro* methylation assays using as source of substrate protein extracts from *Vcpkmt* ^*-/-*^ mouse organs or three human *Vcpkmt* ^*-/-*^ cell lines. The results show that VCPKMT is a highly specific enzyme, and suggest that VCP is its sole substrate. The *Vcpkmt* ^*-/-*^ mice were viable, fertile and had no obvious pathological phenotype. Their body weight, life span and acute endurance capacity were comparable to wild-type controls. Overall the results show that VCPKMT is an enzyme required for methylation of K315 of VCP *in vivo*, but VCPKMT is not essential for development or survival under unstressed conditions.

## Introduction

Post-translational modifications (PTM) of proteins create an additional layer of complexity to the proteome and can regulate protein structure, interactions, stability and function [1,2]. Protein methylation is one of the most abundant PTMs and is introduced by methyltransferases (MT) which catalyze the transfer of methyl groups from *S*-adenosyl methionine (AdoMet), predominantly to lysine or arginine residues [2,3]. Protein methylation was initially considered to be a permanent PTM, but recently several demethylases have been identified [4]. The human genome encodes more than 200 MTs and the majority of these are grouped in three large MT classes based on their structure and sequence; seven β-strand, SET and SPOUT [3]. Lysine methyltransferases (KMT) are predominantly found in the SET domain class of MTs.

Valosin-containing protein lysine methyltransferase (VCPKMT), previously named METTL21D, is a seven β-strand methyltransferase and a member of Methyltransferase Family 16, which has recently been shown to encompass several lysine specific MTs [5,6]. VCPKMT has been shown to trimethylate K315 (K315me3) in VCP (see below) [5,6]. For some of the remaining 9 members of this family potential substrates have been identified. HSPAKMT (or METTL21A), one of the closest relatives to VCPKMT, trimethylates a conserved lysine in several Hsp70 proteins [5,7] and thereby influences the chaperone’s affinity for α-synuclein [7]. METTL22 methylates KIN17, a human protein thought to be involved in DNA repair [8], and by that influences its interaction with chromatin [9]. The yeast homolog of METTL18, YIL110W has been shown to methylate a specific histidine in the ribosomal protein RPL3 [10] and is required for a correct 60S subunit assembly [11]. Calmodulin is the target of CAMKMT, another family 16 member [12,13]. FAM86A and its yeast homologue Yij129c were identified as lysine methyltransferases for the eukaryotic elongation factor 2 [14–16]. The only MT Family 16 member localized to mitochondria, METTL20, methylates two adjacent lysines in the electron transport flavoprotein [14,17].

The Valosin-containing protein (VCP) or p97 is a homohexameric ATPase that is involved in a multitude of cellular functions, including endoplasmic reticulum associated degradation, membrane fusion, protein degradation and transcription factor regulation [18,19]. VCP is highly conserved in all eukaryotes and is essential for development in mice [20]. Mutations in VCP are associated with several neurodegenerative diseases [21–23]. However, no mutation at K315 has been reported as a cause for these diseases, and most mutations are located in the N-terminal domain of VCP which is predominantly responsible for protein interactions [24].

In this study, we generated and characterized mice lacking VCPKMT. *Vcpkmt* ^*-/-*^ mice should allow assessment of the physiological consequences of ablating VCP methylation. We show that *Vcpkmt* is expressed in all examined tissues and the protein is predominantly localized to the cytoplasm. We confirm *in vivo* that K315 of VCP is fully trimethylated in mice and that VCPKMT is the only MT responsible for this methylation. In tissues and cells lacking VCPKMT, VCP is completely unmethylated. *Vcpkmt* ^*-/-*^ mice are viable, fertile and show no obvious pathological phenotype.

## Results

### Generation and validation of *Vcpkmt* ^*-/-*^ mice

**Fig 1:**
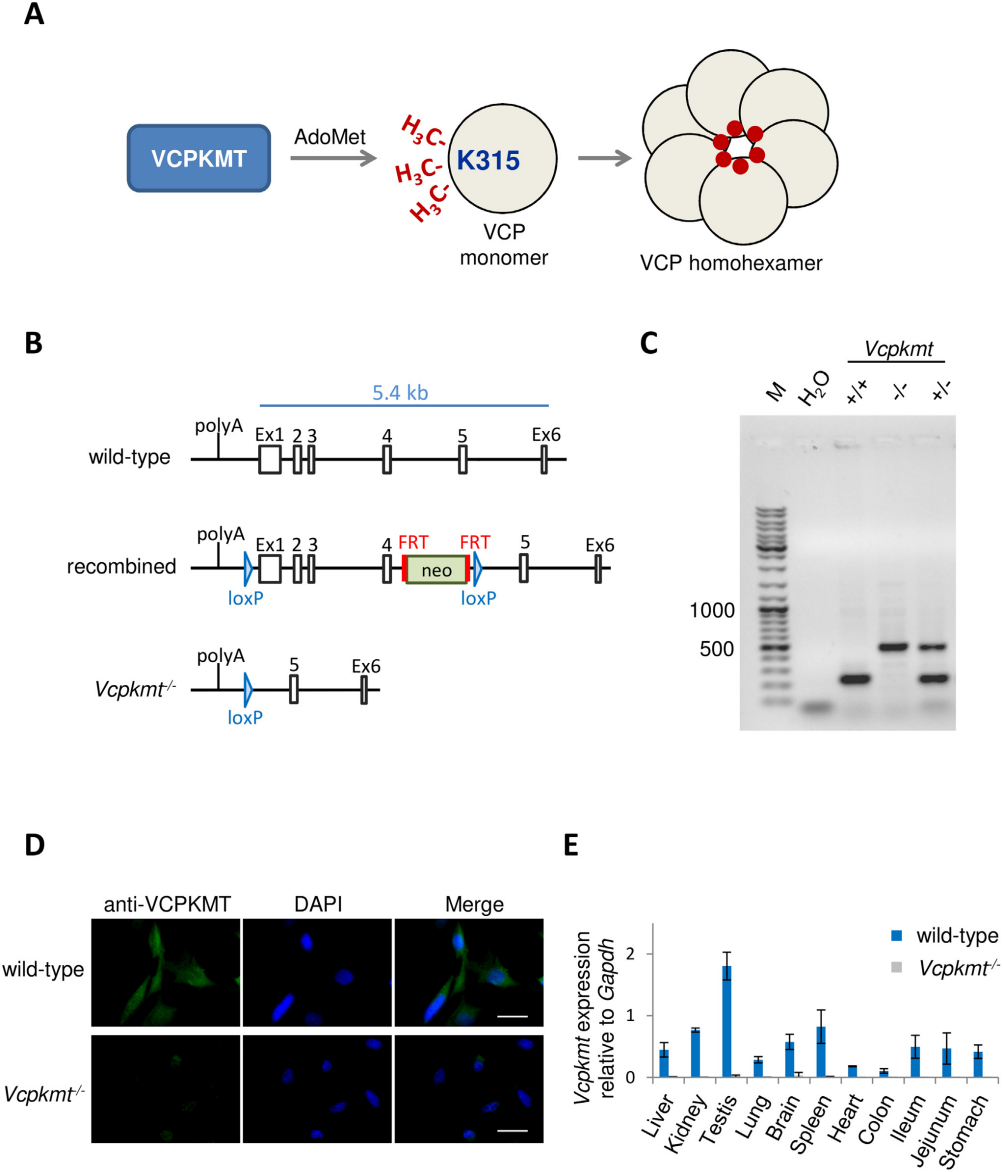
Generation and validation of *Vcpkmt* knockout. (**A**) Schematic overview of K315 trimethylation of VCP monomer by VCPKMT. AdoMet = S-adenosyl methionine (**B**) Targeting of the *Vcpkmt* gene. Diagram shows the endogenous murine *Vcpkmt* locus, the position of the targeted gene and the recombined gene. (**C**) Representative genotyping result from *Vcpkmt* wild-type (+/+), knockout (-/-) and heterozygous (+/-) mice. (**D**) Immunostaining for VCPKMT in wild-type and *Vcpkmt* ^*-/-*^ mouse embryonic fibroblasts. DNA counterstain with DAPI. Scale bars 10 μm. (**E**) The gene expression of *Vcpkmt* relative to *Gapdh* in various murine tissues measured with quantitative RT-PCR. n = 3 mice, means ± SD.

VCPKMT is a MT that uses AdoMet to trimethylate K315 in the VCP monomer. Trimethylated VCPs are then assembled to a fully methylated homohexameric complex (Fig 1A). We generated a conditional knockout mouse for *Vcpkmt* by introducing loxP sites flanking exons 1 and 4 of the *Vcpkmt* gene (Fig 1B) and thereby deleting more than 82% of the *Vcpkmt* coding region. After breeding these recombined mice with constitutively Cre recombinase-expressing mice, complete removal of genome sequences spanning exon 1 to 4 of *Vcpkmt* was confirmed. The DNA from all offspring was analyzed by PCR with specific primers, as outlined in the methods section, to determine their genotype (Fig 1C and S1 Fig). We generated mouse embryonic fibroblasts (MEFs) from wild-type and *Vcpkmt* ^*-/-*^ mice. Cells lacking VCPKMT had proliferation rates and growth characteristics comparable to wild-type cells (S2 Fig). VCPKMT depletion in *Vcpkmt* ^*-/-*^ mice was verified with immunofluorescence with VCPKMT anti-serum (Fig 1D). In the wild-type MEFs VCPKMT is predominantly localized to the cytoplasm (Fig 1D).

### VCPKMT is expressed ubiquitously

Further we wanted to analyze the gene expression of *Vcpmt* in various organs. To this end we isolated RNA from several tissues of 6 months old wild-type and *Vcpkmt* ^*-/-*^ mice and performed quantitative RT-PCR to measure the mRNA levels. The results show that *Vcpkmt* is ubiquitously expressed and that the highest expression is in testis (Fig 1E). Overall, the variation of *Vcpkmt* expression across tissues was not very high. In *Vcpkmt* ^*-/-*^ mice, *Vcpkmt* mRNA expression was untraceable (Fig 1E). PCR analysis with primers specific for different exons of *Vcpkmt* confirmed lack of expression, indicating a complete removal of both reported isoforms of the protein (data not shown).

### VCP methylation is abolished in *Vcpkmt* ^*-/-*^ mice

**Fig 2:**
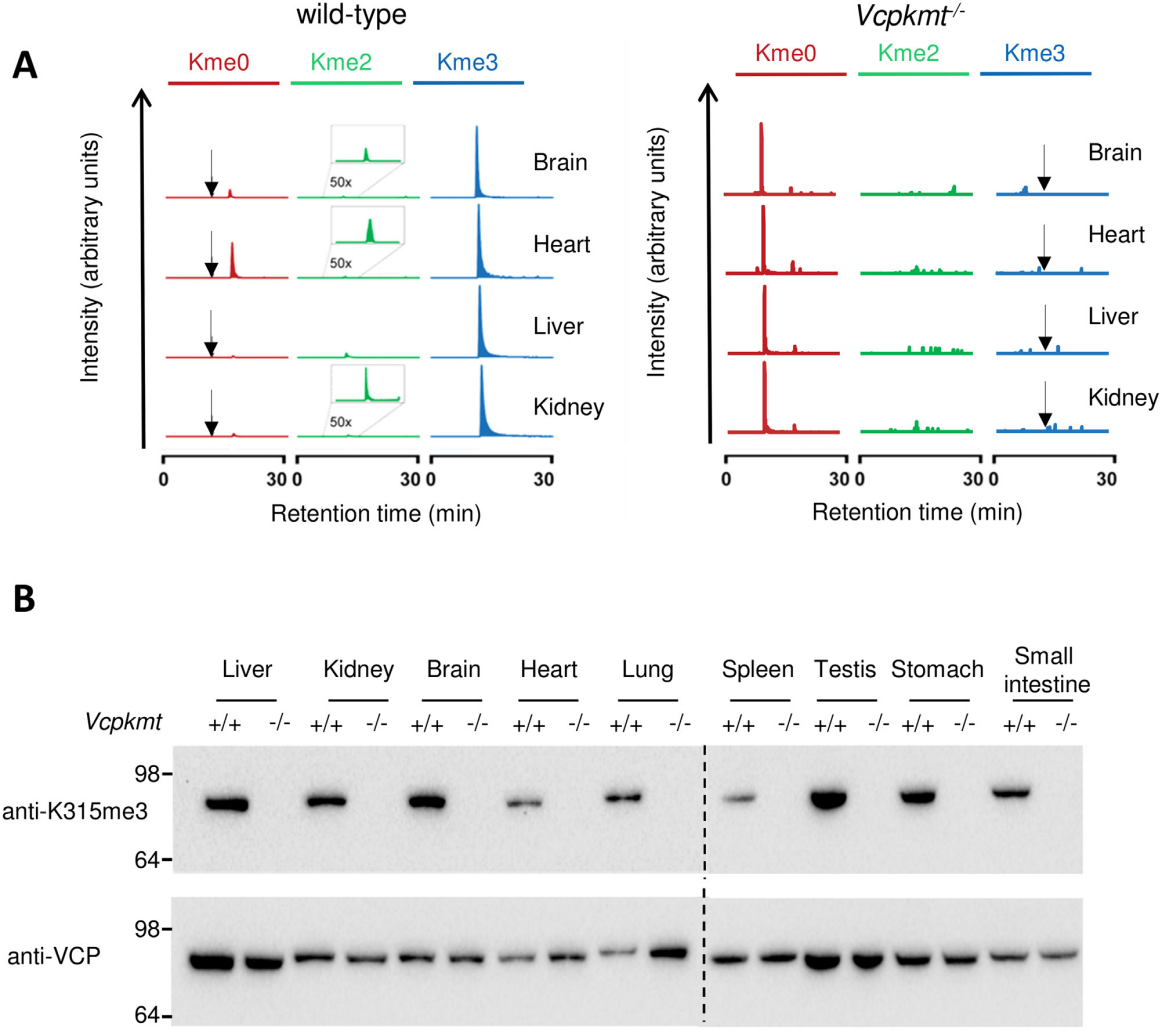
VCP K315 trimethylation is abolished in *Vcpkmt* ^*-/-*^ mice tissues. (**A**) Mass spectrometric analysis of VCP isolated from wild-type (*Vcpkmt* ^*+/+*^) and *Vcpkmt* ^*-/-*^ mouse tissues. Extracted ion chromatograms corresponding to un-, di- and trimethylated K315 in Arg-C-generated peptide VCP (314–322) are shown. For each tissue the intensity is normalized to the Kme3 signal. Expected elution time of Kme0 (left panel) or Kme3 (right panel) peptides are indicated with an arrow. The data for wild-type tissues have been published previously [6]. (**B**) Immunoblotting for K315me3-VCP in various wild-type and *Vcpkmt* ^*-/-*^ tissue protein extracts. 30 μg of whole cell extracts are loaded. Total VCP is shown as a loading control.

**Fig 3:**
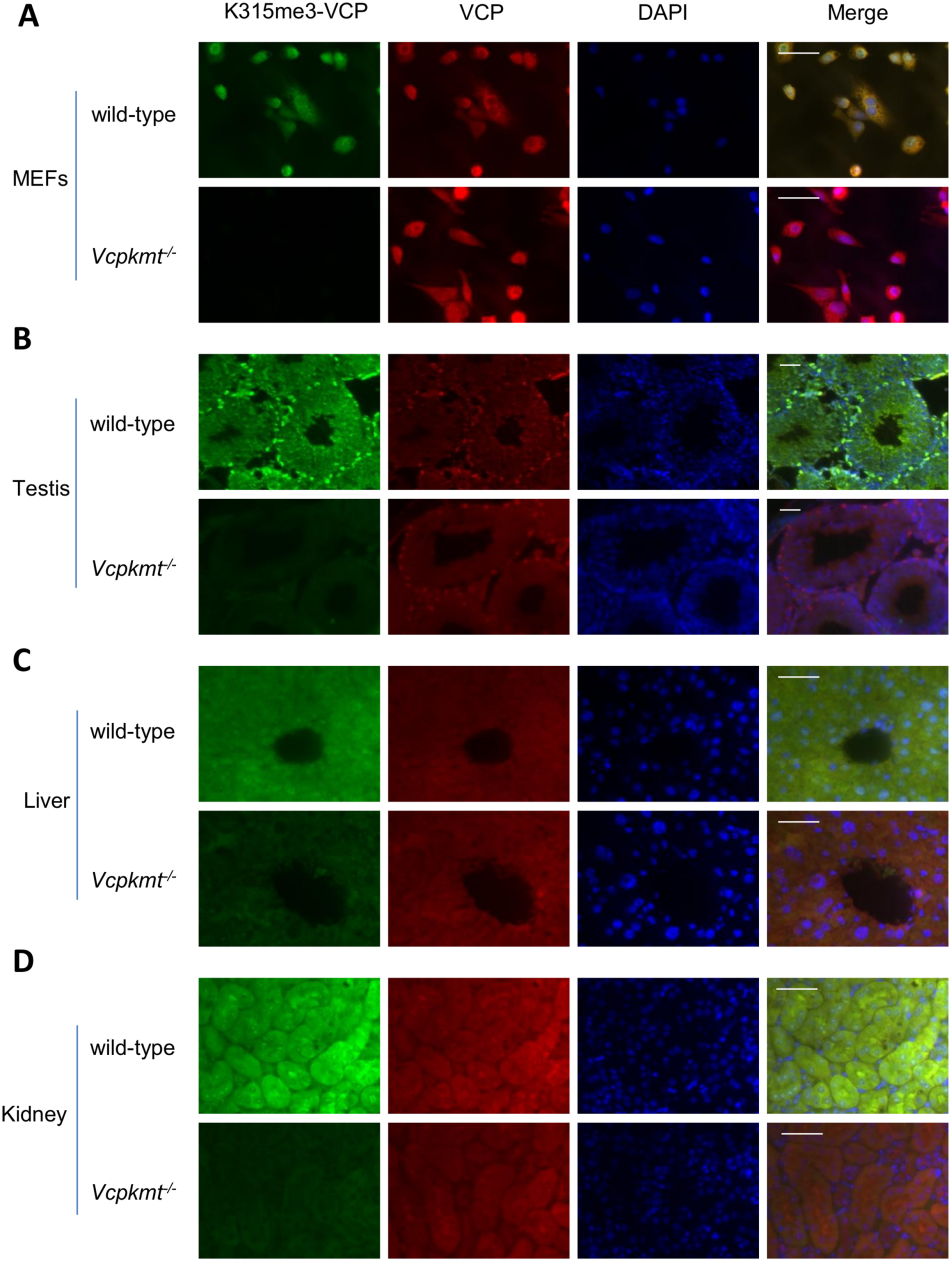
Immunostaining of K315me3-VCP in cells and tissues of wild-type and *Vcpkmt* ^*-/-*^ mice. Immunostaining of K315me3-VCP (green), total VCP (red) and DAPI DNA counterstain (blue) of wild-type and *Vcpkmt* ^-/-^. Scale bars 20 μm. (**A**) Cultured mouse embryonic fibroblast (MEFs). (**B**) Semniferous tubuli of testes. (**C**) Hepatocytes with a central vein. (**D**) Kidney cortex.

Next we wanted to test if VCP is methylated *in vivo* and if this methylation is mediated solely by VCPKMT or if maybe other methyltransferases could compensate the loss of VCPKMT in mice. We immunoprecipitated total VCP from different mouse tissues, ran it on an SDS-PAGE-gel, followed by excision of the relevant band, in-gel digestion with Arg-C, and analysis of the resulting peptides by mass spectrometry (Fig 2A). The presence of VCPKMT in wild-type mice results in almost exclusively trimethylated VCP, while only about 0.5% of the VCP molecules are dimethylated. In the four *Vcpkmt* ^*-/-*^ tissues analyzed neither mono-, di- or trimethylated VCP could be detected (Fig 2A right panel). To further study the VCP-K315 methylation status in different tissues we generated a K315me3-VCP specific antibody, which only recognizes a trimethylated peptide containing the sequence around VCP-K315 but not the matching unmodified sequence (S3 Fig). We then used this K315me3-VCP antibody to analyze by Western blotting the VCP methylation in whole cell protein extracts from various tissues. In all the organs examined, the methylation signal was present in the wild-type, but completely absent in the extracts from *Vcpkmt* ^*-/-*^ mice (Fig 2B). An antibody recognizing VCP, regardless of its methylation status, was used as a loading control and VCP extent did not vary between wild-type and *Vcpkmt* ^*-/-*^ mice (Fig 2B). To further establish the distribution of K315me3 *in vivo*, we generated primary MEFs and analyzed K315me3 by immunofluorescence staining (Fig 3A). K315me3 could only be detected in the wild-type and showed an almost perfect co-localization with the total VCP. In addition we investigated the methylation status of VCP in mouse tissues. For that purpose we used co-immunostaining of VCP and K315me3-VCP in formalin fixed, paraffin embedded tissue sections. In testis (Fig 3B), liver (Fig 3C) and kidney (Fig 3D) it is clearly visible, that in *Vcpkmt* ^*-/-*^ mice the trimethylation signal is severely diminished. This difference is not as striking as in the primary MEFs, probably because of the different fixation methods. In addition we analyzed the K315me3 distribution in spleen and lung, which gave comparable results (S4 Fig).

### VCPKMT specifically methylates VCP

**Fig 4:**
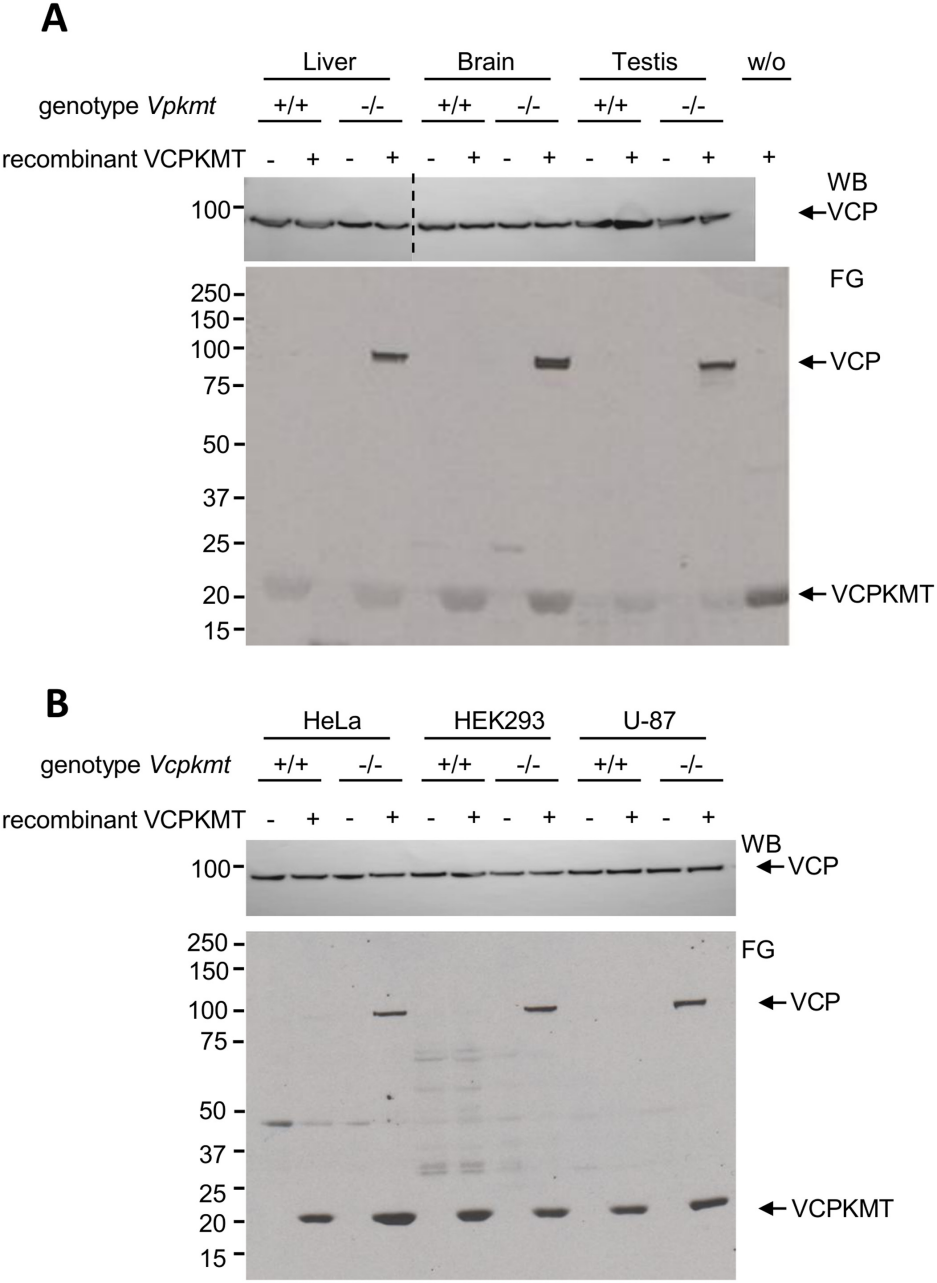
VCPKMT-mediated *in vitro* methylation using extracts from *Vcpkmt* ^*-/-*^ mice tissues and human *Vcpkmt* ^*-/-*^ cell lines. Cell extracts were incubated with recombinant VCPKMT in the presence of [^3^H]AdoMet, proteins separated by SDS-PAGE, and then transferred to a PVDF membrane, which was then subjected to fluorography (FG, lower panels). Extracts were from (**A**) liver, brain and testis of wild-type and *Vcpkmt* ^*-/-*^ mice and (**B**) human wild-type and *Vcpkmt* ^*-/-*^ cell lines. Western Blot (WB) against VCP serves as loading control (upper panels). w/o = without protein extract.

We wanted to test the specificity of VCPKMT by an *in vitro* methylation assay, with radioactive AdoMet, recombinant VCPKMT, and, as source of substrate, protein extracts from mouse liver, brain or testis, respectively. In these experiments radiolabeled methyl groups were only incorporated into VCP when *Vcpkmt* ^*-/-*^ extracts were used (Fig 4). This shows that VCP is not amenable to methylation in wild-type extracts, likely because K315 is already fully trimethylated. In addition to the specific VCP-methylation, the VCPKMT-dependent band at approximately 22 kDa (Fig 4A) indicates most likely an auto-methylation of VCPKMT, which has been shown before [6]. The specificity of the VCPKMT enzyme was also confirmed in three human previously described knockout cell lines generated with zinc finger nucleases (Fig 4B) [6].

### *Vcpkmt* ^*-/-*^ mice lack an obvious phenotype

**Fig 5:**
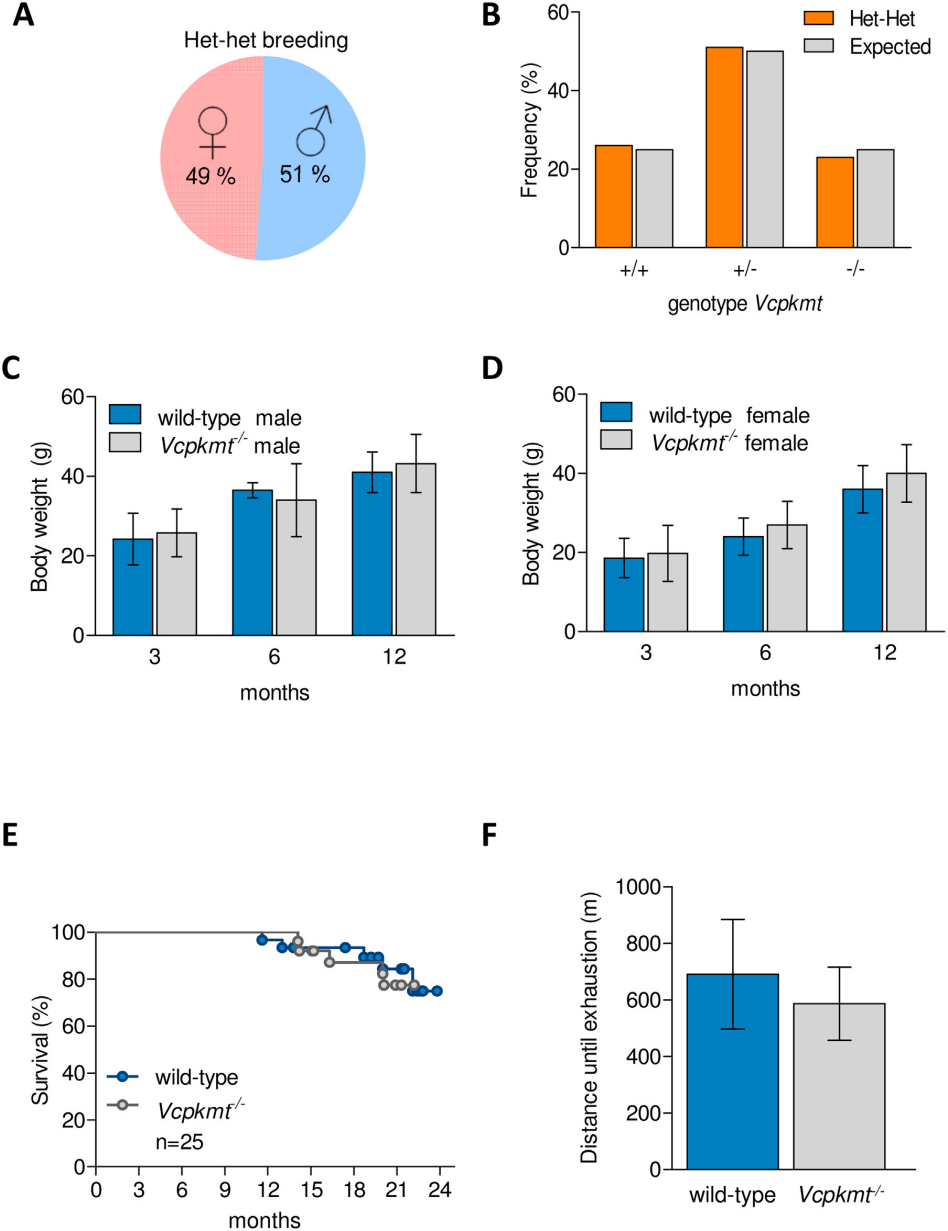
Breeding and survival of *Vcpkmt* ^-/-^ mice. Basic phenotypes of *Vcpkmt* ^-/-^ mice. (**A**) Sex-ratio of an interbred of *Vcpkmt* ^+/-^ (het) mice. (**B**) Genotype distribution of het-het breedings compared to an expected Mendelian distribution. +/+ = wild-type, +/- = heterozygous, -/- = knockout. n = 49. Average body weight per genotype of male (**C**) and female (**D**) mice with the age of 3, 6, 12 months. Means ±SEM. n = 6–8 mice. (**E**) Kaplan-Meyer-Plot of the survival of wild-type and *Vcpkmt* ^*-/-*^ mice monitored over 24 months. n = 25 mice per genotype. (**F**) Running distance on a treadmill during an acute exhaustion assay. Means ±SEM. n = 6–7 male mice, age 8–10 weeks.

*Vcpkmt* ^*-/-*^ mice develop normally with no obvious phenotype and they appear to be healthy without any apparent difference from wild-type controls. When heterozygous mice (HET) were interbred, the sex-ratio (Fig 5A) and the distribution of the obtained genotypes (Fig 5B) followed the expected Mendelian distribution. Also, the average litter size was in the range of the mouse strains used (S5 Fig). This shows that the knockout mice are viable, fertile and have no defect in embryonic development. The body weight in males (Fig 5C) and females (Fig 5D) of *Vcpkmt* ^*-/-*^ mice was not significantly different from wild-type mice. In addition, the survival of the *Vcpkmt* ^*-/-*^ mice in an ageing study and the detected causes of death were similar to the wild-type mice (Fig 5E). Furthermore, we tested the endurance with an acute exhaustion protocol on a mouse treadmill. There was no difference in running capacity between wild-type and knockout male mice at an age of 8–10 weeks (Fig 5E).

## Discussion

In this paper we have described the generation and characterization of *Vcpkmt* ^*-/-*^ knockout mice. We show that VCPKMT is essential for the generation of K315me3-VCP and that this modification is completely absent in VCPKMT deficient cells or tissues.

In vitro methylation assays with cell extracts showed methylation of a single substrate, with an apparent molecular weight corresponding to that of VCP, indicating that VCP is a highly specific enzyme. Interestingly, two studies independently identified VCP as a substrate of VCPKMT, although different approaches (yeast two hybrid screening and tandem affinity purification) were used to identify VCP as a binding partner of VCP-KMT [5,6]. Taken together, this suggests that VCP is the only biologically relevant substrate of VCPKMT. However, since VCP is very abundant, and since these approaches are more likely to retrieve high-abundance proteins, one can’t exclude the possibility that other substrates do exist.

In mammals, more than 20 protein lysine MTs with over 60 non-histone substrates and different target lysines in these substrates have been identified [2]. Some of these MTs also methylate histones and for these MTs the knockout mouse models show embryonic lethality or severe developmental defects. For example DOT1L, histone MT belonging to the 7-β-strand class, and SMYD2, which methylates histones and HSP90 in the cytosol, are essential for embryonic development [25,26]. But mice lacking SET7/9, the most promiscuous non-histone MT, are viable [27,28]. Very recently, it has been shown that Calmodulin lysine methyltransferase knockout mice have reduced body growth, somatosensory development deficiency and impaired mouse brain function [29]. Here in this study, we show that the absence of the VCPKMT protein and therefore K315me3-VCP is not associated with any obvious pathological phenotype. The mice were viable, fertile, and did not have a shortened lifespan. It will be interesting to see if the absence of related MTs or combinations of those will have a more severe phenotype. Human METTL23, a MT Family 16 member without a yet described target, is associated with intellectual disability and interacts with the transcription factor GABPA [30,31]. Taken together this suggests that protein lysine methylation of non-histone substrates is not crucial for development but is involved in the regulation of molecular pathways and/or protein interaction networks.

VCP (also called p97) is a highly conserved member of the AAA+-ATPases (ATPases associated with various activities) family, involved in several cellular pathways including protein degradation, endoplasmic reticulum-associated degradation, cell cycle regulation, membrane fusion and DNA damage response [18,24,32]. VCP is a very abundant protein and has more than 30 interaction partners which mostly interact with the N-terminal domain of VCP [24]. Mutations in VCP have been linked to severe degenerative disorders including inclusion body myopathy with frontotemporal dementia (IBMPFD) [23] and amyotrophic lateral sclerosis [21]. The pathogenesis of IBMPFD, which mainly affects muscle and brain, is attributed to autosomal-dominant amino acid substitutions, which most likely cause a shift in the ATPase activity of VCP [33,34]. Hitherto, the physiological role of K315me3 in VCP has not been established. Also the effect of K315me3 on the ATPase activity of VCP is not yet clear. VCP consists of two ATPase domains (D1 and D2) and an N-terminal adaptor domain [19,32]. The K315 methylation site is close to the Walker B motif of the D1 domain. Cloutier et al. showed in an *in vitro* phosphate release assay that a truncated VCP, which is trimethylated by recombinant VCPKMT, has a diminished ATPase activity [5]. But *in vivo*, the VCPKMT deficiency in human cell lines did not influence the overall ATPase activity of endogenous VCP when compared to wild-type cells [6].

Cloutier et al. identified the Tether containing UBX domain for GLUT4 protein (TUG), which is encoded by the *Aspscr1* gene, as an interaction partner of VCPKMT [5]. TUG retains GLUT4 transporters in the cytoplasm; insulin triggers TUG-cleavage and subsequent localization of GLUT4 to the cell membrane and muscle sarcolemma [35,36]. *In vitro* methylation of VCP by VCPKMT can be stimulated by the addition of TUG or its C-terminal fragment [5]. TUG is capable of disassembling the VCP hexamer into monomers [37] that are preferentially methylated by VCPKMT [6]. Hence the TUG-dependent VCPKMT stimulation could be an indirect effect of TUG’s VCP-disassembling activity. It has been speculated that TUG and VCPKMT may regulate ubiquitin-dependent sorting of GLUT4 via VCP methylation [38]. GLUT4 is important for exercise tolerance by increasing the glucose uptake into the muscle [39]. We show that *Vcpkmt* ^*-/-*^ mice have no attenuated acute running capacity compared to wild-type mice. Therefore, it is unlikely that VCP methylation stimulates GLUT4 localization to the sarcolemma of the muscle. Such a stimulation would enhance glucose uptake [40,41], which would then boost muscle contractions and thereby acute exercise capacity [42].

The full trimethylation and the absence of mono- and dimethylation of VCP observed in the wild-type mice (Fig 2A) suggests that there is no dynamic up- and down-regulation of VCP-methylation. If VCP-methylation levels would be regulated by demethylases or via VCPKMT activity changes, one would expect to find varying levels of K315me3 in different tissues or detect the intermediate methylation states more frequently. The complete trimethylation of VCP in the wild-type situation makes it also unlikely that the K315me3 competes with other post-translational modifications, like actetylation or ubiquitination, which can occur on lysines as well [2]. Previously, K315 has been identified as a site of acetylation [43], but this may be a technical artifact. Acetylation (ΔM 42.0106) and trimethylation (ΔM 42.0470) cause a very similar change in protein mass and therefore are often difficult to distinguish in mass spectrometric analysis.

In this study we show in murine cells that VCPKMT is located to the cytoplasm and co-localizes with VCP. This is in accordance with a previous study that shows that in human cell lines ectopically expressed VCPKMT localizes predominantly to the cytoplasm and shows almost perfect co-localization with VCP [5].

Recently, it has been shown that VCPKMT is involved in cancer metastasis formation [44]. After injecting VCPKMT-overexpressing cells in mice, the metastatis formation in lymph nodes was enhanced, but the primary tumor size was reduced. Furthermore, some human cell lines deficient for VCPKMT showed a reduced migration and invasive potential [6]. In the same study, some of the human knockout cell lines had a slower cell proliferation, but in our analysis the primary MEFs did not change their growth characteristics according to the VCPKMT status. Further studies will have to elucidate if *Vcpkmt* ^*-/-*^ mice have an altered cancer metastasis formation.

Overall, our results show that despite the complete loss of K315me3 in VCP in *Vcpkmt* ^*-/-*^, the mice appeared to be healthy. Thus VCP methylation by VCPKMT is dispensable for development and survival of mice under unstressed conditions.

## Methods

### Ethical statement

All animal experiments were approved and performed according to the Norwegian Animal Research Authority and local guidelines (Permit numbers 11–3150 & 13–5582). The mice were housed in a minimal disease unit under barrier conditions.

### Generation and genotyping of *Vcpkmt* ^*-/-*^ knockout mice

Generation of conditionally targeted *Vcpkmt* mice was conducted by homologues recombination. Gene targeting vector containing VCPKMT exon 1 to exon 4 was constructed by cloning genomic VCPKMT fragments into the pCR4-TOPO vector (Life Technologies). A 2412 bp sized DrdI/NheI fragment of the short homology arm was inserted into a G139 vector (Genoway) upstream of the LoxP-FRT-Neomycin-FRT (linearized with AvrII/PmeI). A fragment from this construct was inserted into a G194 vector (Genoway) with inserted polylinker (restriction sites AscI-SwaI-XhoI-EcoRV-XmaI-PmeI-FseI-PacI). A synthetic LoxP fragment was inserted into the PflMI site downstream exon 4 of the distal part of the distal long homology arm to generate the distal LoxP site. This insertion destroyed the PflMI site and introduced AvrII, SacI, NheI and SpeI sites. A 2288 bp sized SwaI/XhoI fragment isolated from the long homology arm was inserted into the proximal long homology arm, linearized with SwaI/XhoI. The proximal long arm was joined to the distal long arm by insertion of a 3832 bp sized XhoI/DrdI fragment isolated from the distal long arm into the proximal long arm digested with XhoI/EcoRV. A neomycin positive selection cassette was inserted together with the small homology arm by ligation of a 3602 bp XmaI/EcoRI fragment into the XmaI/PmeI restricted vector. The final targeting vector (13842 bp) was completed by insertion of a Diphtheria Toxin A (DTA) negative selection cassette at the 3’end long homology arm via AscI-NotI. Two LoxP sites were inserted, flanking an inserted neomycin-resistance gene cassette and the exon 1 and exon 4 regions. The targeting vector (13.8 kb targeting fragment) was linearized with Fse I and electroporated into 129Sv/Pas ES cells. ES cells were selected with 200 μg/ml G418 48h after electroporation. Homologues recombination was verified by Southern blot analysis. Positive ES cell clones were selected by cell growth and morphology and injected into C57BL/6J blastocysts. Injected blastocysts were then re-implanted into OF1 pseudo-pregnant females. Chimeric mice were crossed with the B6.Cg-Tg(CMV-Cre)Geno/J Cre-deleter mouse strain (GenOway) to generate heterozygous conventional knockout mice. In this study the Cre-allele was not bred out before the animal experiments.

For the genotyping of the different mice, DNA from ear samples was isolated using the HotShot method [45]. The following PCR mastermix was used: Primers 5 pmol, dNTPs 0.2 mM, MgCl_2_ 3 mM, 1X NH4-buffer, Biotaq-polymerase 0.3U, sample DNA 1 μl in 12 μl total volume. The PCR reaction was conducted with the following conditions: Initial denaturing at 94°C for 240s; 30 cycles with 94°C for 45s, 56°C for 30s, 72°C for 60s; final extension 72°C for 300s. List of primers: Reverse primer 5’-5’-GACTTAAAGGCGACTCGACTAC AACGCTCAGGAAATCGCTAC, WT-forward 5’-GACTTAAAGGCGACTCGACTAC, KO-forward, FLOX-forward 5’-ATGCTCCAGACTGCCTTGGGAAAAG.

### Cell culture

HeLa, U87MG, HEK293 wild-type and *VCPKMT* knockout cells, as described in [6], were cultivated in DMEM supplemented with 10% fetal bovine serum, 1% Glutamax I (Life Technologies) and penicillin/streptomycin. For the mouse embryonic fibroblast generation, the uterine horns of 11.5 dpc female mice were dissected and rinsed briefly with 70% EtOH and PBS. Legs, arms and tail, head and organ sack were removed and the remaining embryo was incubated in trypsin for 15 min at 37C. To generate a cell suspension the tissue is pipetted gently up and down. The cells are then transferred to a 25 cm^2^ tissue culture flask and grown in full MEF-media (DMEM High Glucose with 10% FBS, 1% Pen/Strep and 1mM Glutamine). When confluent, the cells were harvested and frozen in liquid nitrogen.

### Reverse transcription and quantitative PCR

Organs of 6 months old male mice were harvested and snap frozen in liquid nitrogen. RNA was isolated from the frozen liver samples with TRIZOL^®^ (Life Technologies) according to the manufacturer’s manual. One microgram of total RNA and random hexamer primers were used for cDNA synthesis by High Capacity cDNA Reverse Transcription Kit (Applied Biosystems). cDNA samples were diluted and analyzed by quantitative real-time PCR with specific primers. Samples that were not subjected to reverse transcription (“no-RT” control) were analyzed in order to detect potential contamination with DNA. Real-time PCR was performed using Power SYBR^®^ Green qPCR Master Mix (Applied Biosystems) and StepOne Plus Real-time PCR System (Applied Biosystems). Dilutions of a reference cDNA sample were amplified to generate the standard curves. The specificity of PCR products was controlled with agarose gels. The amounts of Gapdh fragments were used to correct for the cDNA yields in the samples. The following primers were used for the qPCR: Vcpkmt-Fw 5’-TGTTACGAACAACGTACAATGGG Vcpkmt-Rv 5’-TGATGGCGGTTTTGGCTTTTT, Gapdh-Fw 5’-AGGTCGGTGTGAACGGATTTG Gapdh-Rv 5’-GGGGTCGTTGATGGCAACA, Actb-Fw 5’-TGCAGCTCCTTCGTTGCCGGT, Actb-Rv 5’- CTTTGCACATGCCGGAGCCGTTGT

### Immunohistochemistry

The various tissues were fixed in 10% neutral buffered formalin and afterwards embedded in paraffin. For immunohistochemistry, tissue sections (4 μm thickness) were deparaffinized, rehydrated and subjected to heat-induced antigen retrieval in Tris-EDTA buffer (10mM Tris, 1mM EDTA, pH 9). The sections were permeabilized with 0.5% Tween 20 in PBS for 15 min at room temperature and blocked with 5% BSA, 5% goat serum in PBST (0.05% Tween) for 90 minutes at room temperature. Slides were then incubated with the primary antibodies overnight at 4°C. After 3 washes with PBST for 5 min, the sections were incubated in the dark with the secondary antibodies. For counterstain DAPI (1 μg/ml) was used and Mowiol 4–88 (Polysciences) was used for mounting. Primary antibodies: mouse anti-VCP (1:250, Abcam, ab11433), rabbit polyclonal anti-K315me3 (1:500, New England Peptides, custom antibody against synthetic peptide H2N-AIAPKRE(3me)KTHGEVERR-OH, double affinity purified), anti-VCPKMT (1:500, serum of rabbits immunized with recombinant human VCPKMT [6]). Secondary antibodies: goat anti-rabbit-Alexa 488 (1:500, Life Technologies), goat anti-mouse-Alexa 594 (1:500, Life Technologies). Images of fluorescently stained sections were acquired using AxioCam MRRev3 camera on an Axio Observer. Z1 microscope (Carl Zeiss).

### Mass Spectrometry

Mass spectrometric analysis of immunoprecipitated VCP was done as described previously [5]. Briefly, isolated VCP-bands were excised from Coomassie-stained SDS-PAGE gels, and incubated with endoproteinase Arg-C (Roche). Extracted peptides were subjected to nanoflow on-line liquid chromatographic MS analysis.

### Western Blot

Approximately 100 mg of each tissue was homogenized in 500 μL of Radioimmunoprecipitation assay (RIPA) buffer (50mM Tris HCl pH 8, 150 mM NaCl, 1% NP-40, 0.1% SDS, 1X protease inhibitor Complete (Roche), 0.1 mM PMSF) by using the FastPrep-24 System (MP Biomedicals) according to the manufacturer’s instruction. Whole cell extracts (WCE) were collected after centrifugation for 30 min at 16,000 × g / 4°C and stored at −80°C until analyses. Then 20 μg of WCE was separated on an SDS-PAGE (Novex^®^ 12% Bis-Tris-Gel, Life Technologies) and blotted on a nitrocellulose membrane with the Trans-Blot-Turbo System (Biorad). The membrane was then blocked for 60 minutes with 10% skim milk in PBST (0.05% Tween 20 in PBS) and incubated with the corresponding primary antibody. After extensive washing with PBST, the membrane was incubated with an HRP-conjugated secondary antibody and detected with SuperSignal West Dura substrate (Thermo Fisher). The antibodies used were: α-K315me3-VCP (1:1000, rabbit, custom antibody against the sequence H2N-AIAPKRE(3me)KTHGEVERR-OH, New England Peptides), α-VCP (1:2000, mouse, ab11433, Abcam), HRP-conjugated goat anti-mouse (1:20000, Sigma), HRP-conjugated goat anti-rabbit (1:40000, Abcam)

### Dot-blot assay

The VCP-peptides in the four different methylation states (sequence: H2N-AIAPKREK(me0/1/2/3)THGEVERR-OH) and an unrelated sequence control peptide (sequence: H2N-CTNWDDMEKIWHHTFY) were dissolved in 50 mM Tris (pH 7.5) plus 0.1 mg/ml BSA. Then this solution (with 2–25 pmol peptide) was spotted on a nitrocellulose membrane and dried. The membrane was blocked for 30 min with 5% milk in TBS-T (20 mM Tris-HCl, 150 mM NaCl, pH7.5, 0.05% Tween 20). The primary antibody was then added (α-K315me3-VCP 1:500) for 45 min at RT. After washing 3 times with TBS-T the membrane was incubated for 30 min with a HRP-conjugated secondary antibody (1:40000, Abcam). After washing 3x with TBS-T the signal was detect with an ECL reagent.

### In vitro methylation assay

*In vitro* methylation was done as described in [6]. Briefly, mice organ tissues (Homogenized with Dounce Homoginiser) and human cells (wild-type or *Vcpkmt* ^*-/-*^ of HeLa, HEK293 and UO87MG respectively [6]) were lysed in lysis buffer (50mM Tris-HCl pH7.6, 100mM NaCl, 5% Glycerol, 1% Triton-X 100, 1mM DTT) and 10μg of these lysates were treated with 100 pmol recombinant VCPKMT, 13μM of [^3^H]-AdoMet in 50μl of MT buffer (50mM Tris-HCl, 50mM KCl and 5mM MgCl_2_) at 37°C for 1 hour. These samples were used to run the western blot for VCP quantification and then the same blots were used for fluorography. Blots were dried and sprayed three times with En3Hance (Perkin Elmer) and exposed to Kodak BioMax MR film at -80°C.

### Cell proliferation assay

50000 cells of wild-type and knockout primary MEFs were plated in duplicates in a 6-well plate (day 0) for each later time point. The cells were then at the analyzing time points (24, 48 and 72 hours after plating) washed, trypsinized and resuspended. Cell counting was performed with a Countess^®^ automated cell counter (Life Technologies).

### Treadmill acute exhaustion assay

Acute exhaustion was tested on a motorized mouse treadmill (LE8710, Panlab) with an electric grid as a motivational stimulus. On the day before the experiment the mice were acclimatized to the treadmill for 5 min without movement, afterwards the training protocol with 5 min at 3 m/min, 5 min with 6 m/min and 5 min with 9 m/min was conducted. On the following the day the acute exhaustion performance was tested with the following settings: 5 min with 6 m/min, then 2 min with 9 m/min and subsequent increasing the speed by 3 m/min every 2 min up to 30 m/min. The mice were then run with the final speed and an incline increase by 5° every 10 min until exhaustion. Exhaustion was defined by entering the electric grid (0.2 mA) for more than 3 sec, two times on the grid for more than 1 sec or staying on the grid for more than 50% of the time.

## Supporting Information

S1 Fig Schematic overview of Vcpkmt knockout. Targeting of the *Vcpkmt* gene. Diagram shows the wild-type murine *Vcpkmt* locus, the position of the targeted gene and the completed knockout. The positions of the forward (fw) and reverse (rev) genotyping primers are indicated.
(PDF)

S2 Fig Cell proliferation of primary *Vcpkmt* ^*+/+*^ and *Vcpkmt* ^*-/-*^ mouse embryonic fibroblasts. 50000 cells of 2 different wildtype and knockout cell lines were plated in duplicates (day 0) and counted for 3 consecutive days (days 1–3). n = 2 (2 duplicates per experiment). Means +/- S.D.
(PDF)

S3 Fig Dot-blot against K315me3 with methylated peptides to show antibody specificity. Dot-blot of increasing concentrations (0, 2, 5, 15 and 45 pmol respectively) of unmethylated (K315), monomethylated (K315me1), dimethylated (K315me2) and trimethylated (K315me3) peptides of VCP region covering K315 stained with the anti-K315me3-VCP antibody. Control is a peptide with an unrelated sequence in the same concentrations.
(PDF)

S4 Fig Immunostaining of formalin-fixed paraffin tissue sections. K315me3-VCP (green), total VCP (red) and DAPI DNA counterstain (blue) of wild-type and *Vcpkmt* ^-/-^. Scale bars 20 μm. **A**. Lung tissue **B**. Spleen
(PDF)

S5 Fig Average litter size of different mice breedings. Average litter sizes of heterozygous (+/-), wild-type (+/+), knockout (-/-), floxed (fl/fl) breedings. n = 7–11 litters. Average litter sizes for the background strains are taken from The Jackson Laboratory.
(PDF)
