# Supplementary material for: Lysine Methylation of the Valosin-Containing Protein (VCP) Is Dispensable for Development and Survival of Mice
Source: PLoS One. 2015 Nov 6;10(11):e0141472. doi: 10.1371/journal.pone.0141472 (PMC4636187; doi:10.1371/journal.pone.0141472)
Supplement: S1 Fig — Targeting of the Vcpkmt gene. Diagram shows the wild-type murine Vcpkmt locus, the position of the targeted gene and the completed knockout. The positions of the forward (fw) and reverse (rev) genotyping primers are indicated. (PDF) [file pone.0141472.s001.pdf]

S1 Fig

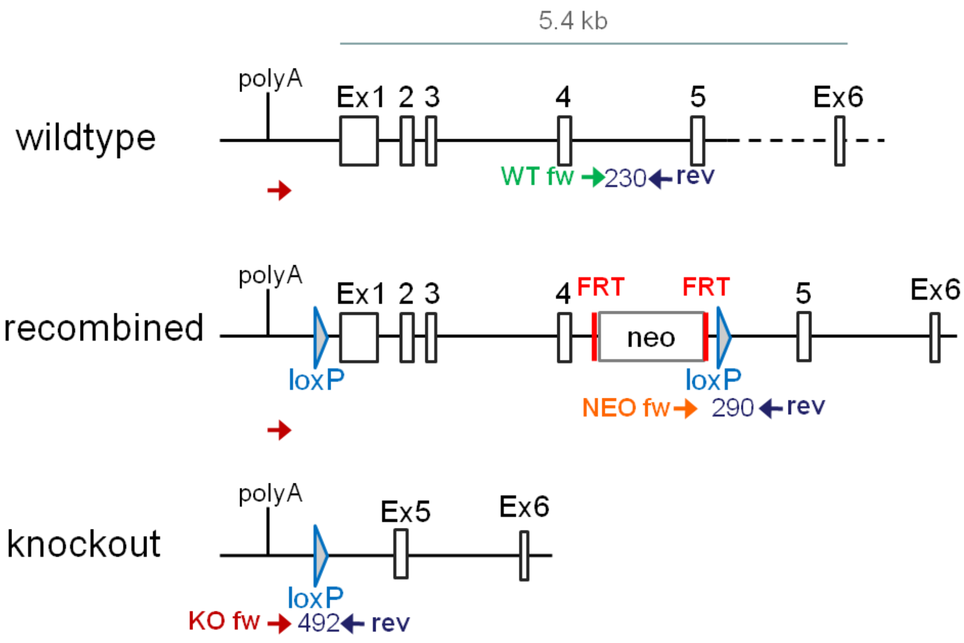

S1 Fig – Schematic overview of *Vcpkmt* knockout. Targeting of the *Vcpkmt* gene. Diagram shows the wild-type murine *Vcpkmt* locus, the position of the targeted gene and the completed knockout. The positions of the forward (fw) and reverse (rev) genotyping primers are indicated.
