# Supplementary material for: Lysine Methylation of the Valosin-Containing Protein (VCP) Is Dispensable for Development and Survival of Mice
Source: PLoS One. 2015 Nov 6;10(11):e0141472. doi: 10.1371/journal.pone.0141472 (PMC4636187; doi:10.1371/journal.pone.0141472)
Supplement: S2 Fig — 50000 cells of 2 different wildtype and knockout cell lines were plated in duplicates (day 0) and counted for 3 consecutive days (days 1–3). n = 2 (2 duplicates per experiment). Means +/- S.D. (PDF) [file pone.0141472.s002.pdf]

S2 Fig

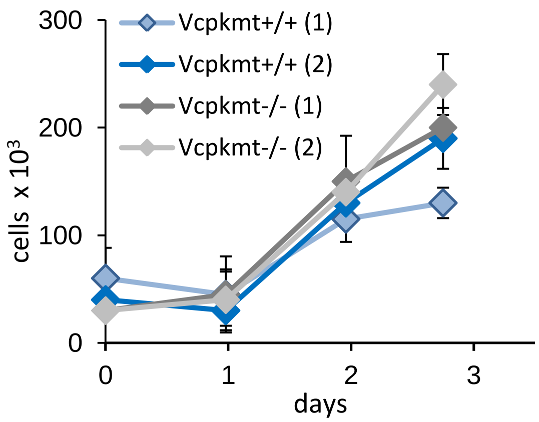

S2 Fig – Cell proliferation of primary *Vcpkmt*$^{+/+}$ and *Vcpkmt*$^{-/-}$ mouse embryonic fibroblasts. 50000 cells of 2 different wildtype and knockout cell lines were plated in duplicates (day 0) and counted for 3 consecutive days (days 1-3). n = 2 (2 duplicates per experiment). Means +/- S.D.
