# Supplementary material for: Lysine Methylation of the Valosin-Containing Protein (VCP) Is Dispensable for Development and Survival of Mice
Source: PLoS One. 2015 Nov 6;10(11):e0141472. doi: 10.1371/journal.pone.0141472 (PMC4636187; doi:10.1371/journal.pone.0141472)
Supplement: S3 Fig — Dot-blot of increasing concentrations (0, 2, 5, 15 and 45 pmol respectively) of unmethylated (K315), monomethylated (K315me1), dimethylated (K315me2) and trimethylated (K315me3) peptides of VCP region covering K315 stained with the anti-K315me3-VCP antibody. Control is a peptide with an unrelated sequence in the same concentrations. (PDF) [file pone.0141472.s003.pdf]

S3 Fig

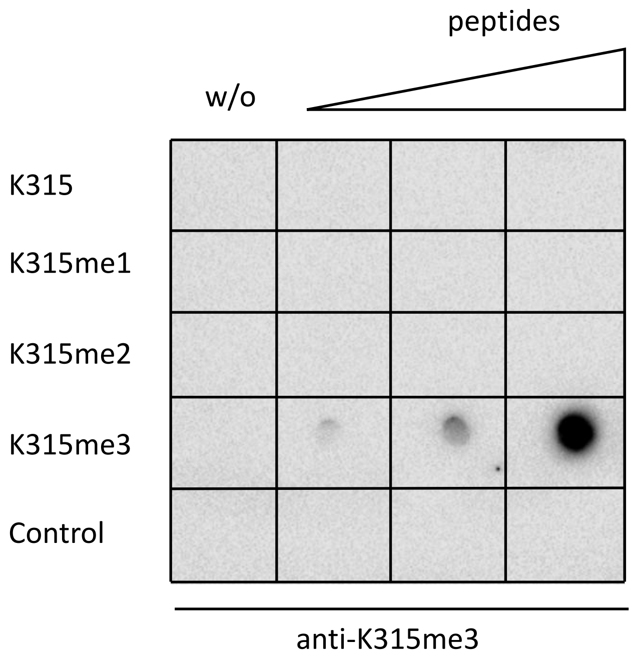

S3 Fig – *Dot-blot against K315me3 with methylated peptides to show antibody specificity*. Dot-blot of increasing concentrations (0, 2, 5, and 25 pmol respectively) of unmethylated (K315), monomethylated (K315me1), dimethylated (K315me2) and trimethylated (K315me3) peptides of VCP region covering K315 stained with the anti-K315me3-VCP antibody. Control is a peptide with an unrelated sequence in the same concentrations.
