# Supplementary material for: Lysine Methylation of the Valosin-Containing Protein (VCP) Is Dispensable for Development and Survival of Mice
Source: PLoS One. 2015 Nov 6;10(11):e0141472. doi: 10.1371/journal.pone.0141472 (PMC4636187; doi:10.1371/journal.pone.0141472)
Supplement: S4 Fig — K315me3-VCP (green), total VCP (red) and DAPI DNA counterstain (blue) of wild-type and Vcpkmt -/-. Scale bars 20 μm. A. Lung tissue B. Spleen (PDF) [file pone.0141472.s004.pdf]

S4 Fig

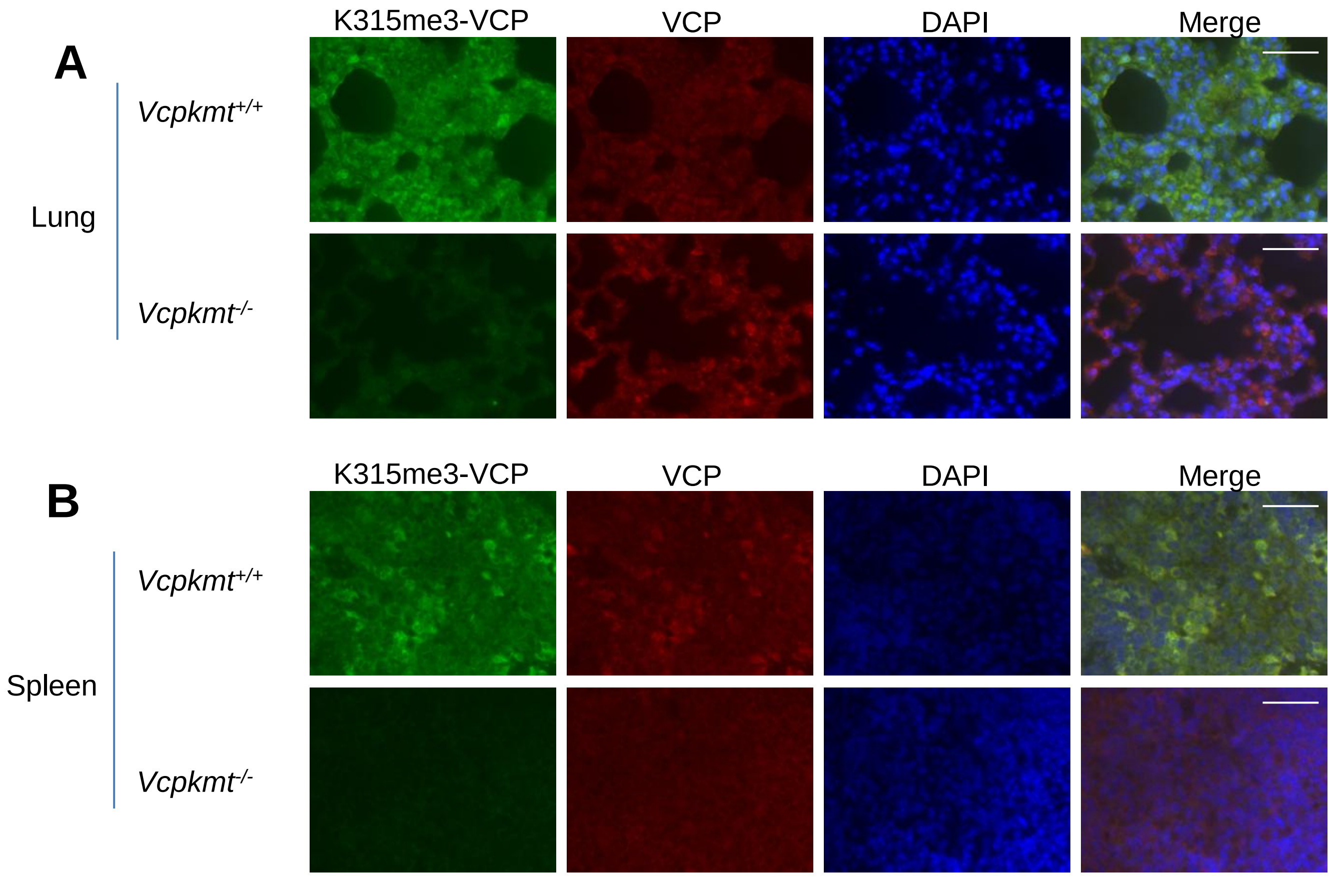

S4 Fig - Immunostaining of formalin-fixed paraffin tissue sections. K315me3-VCP (green), total VCP (red) and DAPI DNA counterstain (blue) of wild-type and *Vcpkmt* $^{-/-}$ *.* Scale bars 20 µm. **A.** Lung tissue **B.** Spleen
