# Supplementary material for: Lysine Methylation of the Valosin-Containing Protein (VCP) Is Dispensable for Development and Survival of Mice
Source: PLoS One. 2015 Nov 6;10(11):e0141472. doi: 10.1371/journal.pone.0141472 (PMC4636187; doi:10.1371/journal.pone.0141472)
Supplement: S5 Fig — Average litter sizes of heterozygous (+/-), wild-type (+/+), knockout (-/-), floxed (fl/fl) breedings. n = 7–11 litters. Average litter sizes for the background strains are taken from The Jackson Laboratory. (PDF) [file pone.0141472.s005.pdf]

S5 Fig

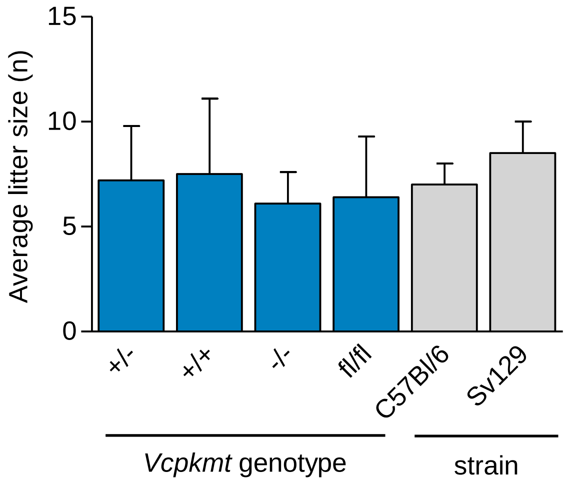

S5 Fig – Average litter size of different mice breedings. Average litter sizes of heterozygous (+/-), wild-type (+/+), knockout (-/-), floxed (fl/fl) breedings. n= 7-11 litters. Average litter sizes for the background strains are taken from The Jackson Laboratory.
